# Valedictory Address of the President of the Illinois State Medical Society

**Published:** 1859-01

**Authors:** C. Goodbrake

**Affiliations:** Clinton


### VALEDICTORY ADDRESS OF THE PRESIDENT OF THE ILLINOIS STATE MEDICAL SOCIETY.

(Delivered at the Annual Meeting in Rockford, June, 1858.)

BY C. GOODBRAKE, M.D., OF CLINTON.

GENTLEMEN OF THE ILLINOIS STATE MEDICAL SOCIETY,—In discharging the last duty pertaining to the station which, by your free-will suffrage, I have had the honor of occupying during the past year, permit me to present for your consideration a few thoughts on some of the causes which, in my opinion, retard medical improvement and the usefulness of our profession, in this our glorious Prairie State.

I know it will be very difficult for me to advance anything new under this head, the ground having been gone over time and again by those more capable and who have more "elegant leisure" to devote to matters of the kind. But as there is scarcely any topic connected with our profession but what has been written and spoken upon by so many, and on multiplied occasions, I hope it will not be deemed arrogant in me to offer a few ideas as they may present themselves to my mind in relation to this subject.

From what observation and *experience* has taught me, I believe that the first and one of the greatest obstacles in the way of medical improvement is the want of a proper or necessary preliminary education on the part of a great many who engage in the study of medicine. Many young men, endowed by nature with excellent minds, and who, if they had undergone a pretty thorough course of mental training, might engage in the study of medicine with great pleasure, and the promise of much success and usefulness, commence it without any educational fitness whatever,—some of them hardly able to write intelligibly or read their own language understandingly. Now it must be quite obvious to every one, who has in any degree profited either by observation or experience, or both, that he who enters upon the study of the science of medicine under these disadvantageous circumstances has indeed a hard task to perform, if his aim is to make himself a respectable and well educated physician.

We will take it for granted that he intends to make himself familiar with the different branches of the study before him, and as a matter of course he will have a double labor to perform from the very outset. In the first place, he must study his text; and secondly, he is compelled, by dint of severe mental labor, to learn to understand the language his author uses. We can all readily perceive that if there were nothing else lost in cases of this kind, it must necessarily involve a great waste of time. It will take the student twice as long as it would if he had been properly educated beforehand to prepare for his college course; the same difficulty will follow him through the whole of his attendance on medical lectures; and he can only hope, by a prolonged course of untiring and undeviating application, to reach the goal for which he has set out.

We must admit that there are some who, by a passionate desire for scientific knowledge and an iron will to overcome all obstacles, will perform almost impossibilities, and finally become pre-eminent in the profession. But while this is true of a few, it is also true, that hundreds of those who commence the study of medicine without a reasonable preparatory education, soon become discouraged by the great disproportion of their mental acquirements and the magnitude of the task before them; and some of them—those who take the most sensible view of the subject—turn their attention to some other calling more proportionate with their educational qualifications; but a majority of them, because they are unable to comprehend either the beauties or usefulness of the science, arrive at the conclusion that all they want is a smattering of the *art of medicine*, and consequently they aspire no higher than to obtain their diploma, and ever after follow their profession as a trade, merely to make money. Of such are those of whom it has been said, that instead of conversing on some scientific subject — the character of the diseases most prevalent, etc., etc.—when they meet with a neighboring physician, they always inquire of him how many dollars he has booked per month. All such men, instead of doing anything that might add to our common stock of medical knowledge, are mere clogs under the wheels of the car of medical progress.

Another great hinderance to medical progress in our western country, according to my way of thinking, is the habit, existing among us to a considerable extent, of sending our students "east to attend lectures." The time may have been when this practice of sending our students "over the mountains" to receive medical instruction was necessary, but that time has gone by; for we can boast of a sufficient number of medical schools, supplied with every material necessary to impart instruction, hospitals included. And our schools are conducted by gentlemen who are as capable, and who are in possession of as high medical attainments as can be found in any portion of our whole country. I am aware that when anything is said on this point, the cry is at once raised against "sectional medicine." They may call it what they please, but I am confident that those who wish to practice medicine on the prairies of the west should receive instruction from men who are acquainted with the peculiar character of the diseases which the student will be called upon to treat when he assumes the duties of a medical practitioner. "That diseases present peculiar features in different localities no one will pretend to deny; and that our western diseases require a different treatment—to a great extent—from those of eastern and mountainous ones, will be corroborated by every candid physician who has received his medical education in the east, and has settled himself to practice his profession in the west. He will tell us that it required considerable time and study before he could familiarize himself with the prevalent diseases of our western country and treat them successfully." Leaving out of the question the risk of the lives of patients and the reputation of the young practitioner, this again is attended with a loss of time; and as time is money in the business world, so time is knowledge in the scientific world.

The next thing to which I will call your attention is a want of the exercise of proper discrimination on the part of too many of our practitioners. If there is any one qualification necessary for the physician to possess, in order to practice with benefit to his patients and honor to himself and the profession, that of thorough discrimination stands pre-eminent. And the want of discriminative powers, or the neglect to exercise them, will continually subject him to upbraidings of conscience, mishaps in practice, and the pity, if not the contempt, of his professional brethren, no matter what his other acquirements may be.

It will be unnecessary for me to mention all those innumerable and awkward mistakes which occur almost daily in the practice of medicine, surgery and obstetrics, from the neglect to discriminate carefully; and I will therefore only endeavor to point out wherein and in what manner this neglect is almost certain to endanger the lives of patients, disgrace individual practitioners, and bring reproach upon the profession.

The great difference of opinion entertained by physicians in regard to many of our most common diseases, has its origin in the want of a close inquiry into the pathology of every disease the physician is called upon to treat, and a careful selection and judicious administration or application of remedies. How often has it been the case that some physician has reported his experience in typhoid fever; given his treatment, and informed us that it proved highly successful,—it acted like a charm!—and in the very next case of this disease some one of his readers was called to see, he pursued the same treatment, without due regard to age, temperament, idiosyncrasy, or peculiar symptoms, which may have accompanied his case, and which were, perhaps, entirely absent in the case reported, and the treatment has proved an entire failure. Perhaps a succession of cases were treated in a similar manner and with a like result, and he then informed us, through the medium of some medical journal, that the reporter of the treatment was in error, that his plan of treating typhoid fever had been subjected to a fair trial in a number of cases, and found most conclusively inadequate to the purpose. The same is true of some who will tell us that they have treated their cases according to some favorite author,—probably according to Wood's Practice—and that the "treatment laid down don't amount to anything." But they forget to inform us that George B. Wood was not present *in propria persona* to make a correct diagnosis in their particular case, and to exercise discrimination in selecting suitable remedies, as they were indicated from time to time throughout the continuance of the diseases. They forget that an author can do no more than to give us the most prominent characteristics of the disease, and point out some general plan of treatment; and that our own discriminative judgment must, in each individual case, teach us what remedies are indicated—when to give a certain one, and when to discontinue it and substitute another.

How many there are who, when they think they have a case of typhoid fever, at once commence a certain routine of remedies as laid down in their text book or some monograph on this disease, and continue to do so until the patient happens to get well or is removed by death from under their jurisdiction. And there are others who set up an opinion of their own in regard to this disease, to the exclusion of all other opinions, no matter from what source; and who have their own peculiar remedy to "cure-all-cases," and administer it indiscriminately in every case they are called to treat. One will tell us that turpentine alone is sufficient to cure any case of typhoid fever; another informs us that brandy and nitrate of silver is all that is required to cure the most stubborn case; and a third intimates that typhoid fever is not so much of a disease after all! and tells us very complacently that all he wants is to get the patient *slightly* salivated at the commencement of the disease, and he is sure to cure every case, and so on almost *ad infinitum*. Each one of these self-willed practitioners have their own peculiar hobby, and give their own specific, to the exclusion of all other remedies.

It must be apparent to every intelligent mind, that these hobbyists are as deep in the mud, as those who follow some routine of treatment laid down by others, without taking the trouble of thinking for themselves, are in the mire. Both of these classes of men must prove unsuccessful practitioners. In the first place, they neglect to attend to that part of their duty which is paramount to all others, namely: to investigate carefully and thoroughly the disease before them, examine every symptom minutely, and spare no pains to arrive at a just conclusion as regards the constitution and peculiarities of their patient; and in the second place, they fail to use a cautious and warrantable discrimination in the selection of their remedies, but either follow up some prescribed rule of medication without considering its applicability to the case under treatment, or mount their favorite hobby, and ride it until too frequently their patient dies, and they ride themselves out of practice and the profession into disrepute.

What has been said of indiscriminate practice in typhoid fever is equally applicable to the treatment of other diseases. I have seen physicians who would give veratrum viride in every case of pneumonia they were called to treat. No matter in what stage of the disease, or what peculiar symptoms the patient was laboring under when first called, if they diagnosed a pneumonic affection, veratrum must be given; no matter if the pulse was hard or soft, full, or threadlike; and whether the skin was hot and dry, or cool and moist; so the pulse was moderately quick, and the patient had a cough, and complained of pain in the breast or side, he must needs take *the* remedy, because some favorite author or professor had recommended it in particular stages of pneumonia and pleurisy. That such practice is unscientific and calculated to do great mischief, all will readily admit; but some of you may doubt whether any one, calling himself a regular physician, could be found who would treat a patient upon such hap-hazard principles, or rather no principles at all. But I can assure you, gentlemen, that my observation leads me to believe that such practice is far too common among us, and that its effects are more prejudicial to the interests of the profession than all the irregular quackery in christendom. "Better by far would it be for the human family that the *armamenta medica* consisted of sugar of milk, or like inert substances, than potent drugs, if the practitioner is unable to distinguish and identify disease," and fails to exercise sound discretion in the selection of remedies.

Another thing to which I would call your attention is the fact that there is too little importance attached, as a general thing, to the jurisprudence of our profession by teachers, students and practitioners; and it is true that some of our most able practitioners neglect it entirely. This is not as it should be. Medical jurisprudence, when rightly considered, holds a very important rank among the sciences; and of all the duties devolving upon the physician, none is more solemn and responsible than his position as a medical witness. And there is nothing that can occur in his practice in either of the other branches of our profession, which could have a tendency to raise him higher in the estimation of the community than an enlightened opinion given in an intricate case before a court and jury; and nothing could tear a good reputation down more suddenly than a great blunder committed under like circumstances. A learned writer has said that "the talents and acquirements of the clergyman and the lawyer are brought to a more searching popular test than those of the physician. The clergyman's public ministrations and the lawyer's efforts at the bar are laid open to the appreciation of all. Their audiences are, to a large extent, competent judges of their efforts, and if they manifest ignorance, stupidity or superficiality, they forthwith fall in public estimation to the low level they deserve." And he then goes on to tell us that the knowledge and abilities of the physician, in the practice of his profession, are not tested in the same direct and open manner. This is true, so far as it regards the ordinary and every day practice of the physician, but there is another quite different position which he is frequently called upon to occupy. When he is called before the judicial tribunals in the capacity of an expert or skilled witness, his knowledge and abilities are tested in the *same direct and open manner* as that of the clergyman's or lawyer's. In this important position,—and perhaps he may be subpœnaed in an unguarded hour to appear before a court and jury to explain the principles and doctrines of our profession, as touching the peculiar case then under investigation—"with the prying curiosity of the bar, the stern scrutiny of the bench, and with the anxious attention of the jury and the bystanders fixed upon him," he may, if he is thoroughly posted in forensic medicine, stand up as an ornament of the profession, "as the high priest of nature, with whom, in her inscrutable laboratory, he is ever familiar." Or he may, through ignorance or indiscretion, ruin his own professional reputation, and bring reproach upon the profession at large. And how often is it the case that physicians, who are good and respectable practitioners, but having neglected this branch of their profession, when they are called to the witness stand, call down upon themselves the pity of the judge, the ridicule of the lawyers, and the jeers of the audience. How necessary then that all, from those who have charge of our medical institutions down to the most obscure country practitioner, should inculcate the necessity of *more attention* to the principles and facts that constitute medical jurisprudence.

Another thing which retards medical improvement, and prevents the profession from becoming as generally useful as it otherwise might be, is a kind of apathy or want of interest exhibited by too many of our medical brethren throughout the state, in our state and county societies. Medical societies are no longer an experiment; the best medical minds in the country all agree that they are instrumental in creating more union in the ranks of the medical fraternity, a greater interest on all subjects connected with medicine; and also, that all members who feel a lively interest in these societies, and are punctual in attending the meetings thereof, are themselves individually benefited. But notwithstanding all this, there are very many who, from some reason or other, stand aloof, and fail to connect themselves with us to aid us with their council and contribute something to our common fund of medical information, and necessarily fail to receive the reciprocal benefit resulting from such a connection. Some indeed will tell us, and even publish it in the journals, that "all the medical talent in the state is not concentrated in the state medical society." Now this is not claimed; but if what our best medical writers say on this subject be true, it is the duty of every physician in the state to connect himself with the state society; and consequently, all these gentlemen have got to do is to fulfil their duty in this respect, and our society will then contain all the medical talent in the state. "'Tis a consummation devoutly to be wished." If we could visit every county in our state, we would find that in those counties where there is no regular organization of the profession, the practice of medicine is followed too much as a mere business, in which the almighty dollar is the leading principle. And here also we would find those old-fashioned jealousies and tricks of trade which make it so very difficult for the people to find the true line of demarkation between regular medicine and quackery. And, on the other hand, in those counties where they have organized medical societies, and have adopted the national code of ethics, we would, as a general thing, find friendly relations, high aspirations, and a spirit of scientific investigation existing among the members of the profession; and they would greet us with the welcome news that empiricism had taken unto itself wings and fled to parts unknown. For the people will, more readily than is generally admitted, distinguish between the truly scientific and gentlemanly physician and the mere pretender.

If what has been said in relation to these several subjects, as impediments to medical improvement, which I have attempted in a brief manner to bring to your notice, be true, it will follow, as a matter of course, that it is our duty to pay due attention to the preparatory education of those who wish to engage in the study of medicine. Admit no young man into our office as a student who is not in posession of a good English education, and sufficient knowledge of the Greek and Latin to enable him to understand the technical names and phrases necessarily used by medical writers and teachers. This will more immediately benefit the student himself, because it will save him a great deal of time and trouble in after years. But it will also contribute to medical progress, for in nine cases out of ten he will make a better physician and take more delight in investigating scientific subjects.

We should encourage our students as far as practicable to attend lectures in or near as possible to the section of country where they expect to locate. It will save them much vexation and trouble during the first years of their practice. Everything else being equal, let us "encourage home manufacture."

We must discountenance hobbyism and the treatment of diseases according to their names, but encourage the study of signs and symptoms of diseases instead of nosological tables; and inculcate by precept and example careful discrimination in the use of remedies.

We should, as a profession, pay more attention to medical jurisprudence. It should be more extensively taught in our medical schools; its importance more strongly impressed upon the minds of students; and we should all give it at least as much attention through life as we do any of the other branches, as it is most certainly not the least in importance.

And last, though not least, let us encourage and support by every means in our power our state and county medical societies. There should be a more universal feeling among our physicians in favor of these organizations. For if rightly supported and their affairs properly managed, they will do more for the furtherance of medical improvement in our State than any other measure or measures that could be instituted for that purpose.

In conclusion, gentlemen, allow me to tender my heartfelt thanks for the courtesy you have so unanimously extended towards me as presiding officer of this society. And I can assure you that your enlightened views on subjects brought before this society for its action, and your good will and kind forbearance, has made the task on my part a very easy and pleasant one.

